# Adult immunization policies in advanced economies: vaccination recommendations, financing, and vaccination coverage

**DOI:** 10.1007/s00038-012-0438-x

**Published:** 2013-01-25

**Authors:** Lauren A. Wu, Elisabeth Kanitz, Julie Crumly, Fortunato D’Ancona, Raymond A. Strikas

**Affiliations:** 1 National Vaccine Program Office, U.S. Department of Health and Human Services, Washington, DC USA; 2 Reparto Malattie Infettive, Istituto Superiore di Sanità, Centro Nazionale di Epidemiologia, Sorveglianza e Promozione della Salute, Rome, Italy; 3 Health Communication and Marketing Group, Health Communication and Technical Training Program, Oak Ridge Institute for Science and Education, Oak Ridge, TN USA; 4 Immunization Services Division, National Center for Immunization and Respiratory Diseases, Centers for Disease Control and Prevention, Atlanta, GA USA

**Keywords:** Adult immunization, Vaccination policy, Vaccination coverage, Vaccine financing, Developed country, Advanced economy

## Abstract

**Objectives:**

While many countries have robust child immunization programs and high child vaccination coverage, vaccination of adults has received less attention. The objective of this study was to describe the adult vaccination policies in developed countries.

**Methods:**

From 2010 to 2011, we conducted a survey of 33 advanced economies as defined by the International Monetary Fund. The survey asked about national recommendations for adults for 16 vaccines or vaccine components, funding mechanisms for recommended adult vaccines, and the availability of adult vaccination coverage estimates.

**Results:**

Thirty-one of 33 (93.9 %) advanced economies responded to the survey. Twelve of 31 (38.7 %) reported having a comprehensive adult immunization schedule. The total number of vaccines or vaccine components recommended for adults ranged from one to 15 with a median of 10. Seasonal influenza (*n* = 30), tetanus (*n* = 28), pneumococcal polysaccharide (*n* = 27), and hepatitis B (*n* = 27) were the most frequently recommended vaccines or components.

**Conclusions:**

Approximately two-thirds of survey respondents do not have a comprehensive adult vaccine schedule, and most do not measure vaccination coverage. We found that a funding mechanism is available for most recommended adult vaccines.

**Electronic supplementary material:**

The online version of this article (doi:10.1007/s00038-012-0438-x) contains supplementary material, which is available to authorized users.

## Introduction

Vaccines are one of the most cost-effective strategies to prevent infectious diseases among children, adolescents, and adults. Globally, vaccination saves an estimated 2–3 million lives each year. (WHO and UNICEF 2005) Many developed countries have robust child vaccination programs, and initiatives, such as the Expanded Programme on Immunisation and the Global Alliance for Vaccines and Immunisation, are helping developing countries build childhood immunization infrastructures and introduce new vaccines. (Levine et al. 2011) In contrast to the importance placed on childhood vaccination, less attention has been paid to adult immunization, even in developed countries with strong public health infrastructures. (Advisory Council for the Elimination of Tuberculosis and Advisory Committee on Immunization Practices 1996; Lang et al. 2011; Levine et al. 2011).

Vaccinating adults against infectious diseases is important because adults can be carriers of infectious diseases to the susceptible, and immunity from childhood immunizations wanes with age, leading to disease outbreaks such as pertussis in the United States. (Zepp et al. 2011) Additionally, many vaccines are most effective among the healthy, and it is good clinical practice to vaccinate healthy adults before they develop chronic conditions that are contraindications for vaccination or make vaccines less effective. Some vaccine-preventable diseases can become more severe with increasing age. Some adults were not vaccinated as children. Last, with increasing globalization, more and more people travel across borders and are susceptible to infectious diseases that are not endemic in their home country.

Successful childhood vaccination programs contain policies recommending vaccination, effective funding mechanisms, and routine vaccination coverage assessment. To better understand adult vaccine policies, we conducted a survey in 2010–2011 of 33 advanced economies around the world as defined by the International Monetary Fund in 2009. (International Monetary Fund October 2009) We asked about adult vaccination schedules, vaccine recommendations, vaccine funding mechanisms, and vaccination coverage availability and estimates. We also investigated the associations of having adult vaccine policies in place with the population size, per capita gross domestic product (GDP), per capita expenditure on health, and out-of-pocket spending on health.

## Methods

To understand the prior literature on vaccine recommendations and vaccine coverage for adults in developed countries, we performed a PubMed/MEDLINE and Internet search for adult vaccine schedules and/or recommendations. The majority of the literature presented influenza vaccination coverage, with a few papers presenting vaccination coverage for other vaccines such as pneumococcal, hepatitis B, and tetanus vaccines. (Bader and Egler 2004, 2009; Beytout et al. 2004; Blank et al. 2009; de Miguel 2006; del Corro et al. 2009; Gavazzi et al. 2007; Kwong et al. 2007; Leggat et al. 2009; Montrieux et al. 2002; Nielsen et al. 2009; Noakes et al. 2006; Pebody et al. 2008; Sammarco et al. 2004; Schenkel et al. 2008; Stuck et al. 2007; van Houdt et al. 2009) We were unable to identify published literature that discussed a comprehensive adult vaccine schedule and adult vaccine financing in developed countries. To determine the denominator, we used the International Monetary Fund’s (2009) classification of advanced economies (*N* = 33). (International Monetary Fund October 2009) This definition includes two special administrative regions (SARs) (Hong Kong SAR and Taiwan Province of China), and 31 countriesin North America, Europe, and Asia (see Table 1 for list of advanced economies).

**Table 1:** Advanced economies surveyed, abbreviations, and validation of data, 2010, survey of adult vaccination policies in advanced economy countries

Advanced economy	Completed survey	Abbreviation	Validated data
Australia	x	AU	
Austria	x	AT	
Belgium	x	BE	x
Canada	x	CA	
Cyprus	x	CY	x
Czech Republic	x	CZ	x
Denmark	x	DK	x
Finland	x	FI	
France	x	FR	x
Germany	x	GE	
Greece	x	GR	x
Hong Kong, SAR China	x	HK	
Iceland	x	IC	
Ireland	x	IR	x
Israel			
Italy	x	IT	x
Japan	x	JP	
Luxembourg	x	LU	
Malta	x	MT	x
Netherlands	x	NL	
New Zealand	x	NZ	
Norway	x	NO	x
Portugal	x	PT	x
Republic of Korea	x	KO	
Singapore			
Slovak Republic	x	SK	x
Slovenia	x	SL	
Spain	x	SP	x
Sweden	x	SD	x
Switzerland	x	SW	
Taiwan, Province of China	x	TW	
United Kingdom	x	UK	x
United States	x	US	

### Instrument

In March–April 2010, we developed a survey instrument that included questions about the availability of an adult vaccine schedule, recommendations for adults to receive specific vaccines or be vaccinated against certain diseases [for 16 vaccines or vaccine components: diphtheria, Bacillus Calmette-Guérin (BCG), hepatitis B, hepatitis A, herpes zoster, human papillomavirus (HPV), measles, meningococcal, mumps, pertussis, poliomyelitis (polio), pneumococcal, rubella, seasonal influenza, tetanus, and varicella], vaccine financing for recommended adult vaccines (public or private as applicable), availability of vaccination coverage estimates among adults, and vaccination coverage for recommended adult vaccines. We asked about vaccine components because countries have different vaccine formulations and combinations, depending on disease epidemiology and products offered by vaccine manufacturers. For questions on availability of private funding, we asked if a legal requirement in the country or SAR for private insurance to pay for vaccination for one or more groups of adults was in place, and, if so, to indicate for which groups of adults (e.g., specific age groups, those with medical conditions, travelers, at-risk employees, the disabled). We also asked if countries recommended other vaccines against vaccine-preventable diseases for adults in addition to the 16 we specifically asked about.

The questionnaire was administered using two formats: a web-based survey and a Microsoft (MS) Word survey sent via email. The web-based survey was pilot tested in four advanced economy countries (France, Ireland, Italy, and United States) in May–June of 2010. After appropriate changes were made based on the pilot test results, the web- and MS Word-based surveys were sent to vaccination policy contacts in each country and SAR in late June 2010.

### Data collection

For the advanced economies in the European Union plus Norway and Iceland (22 countries), we collaborated with the Vaccine European New Integrated Collaboration Effort II (VENICE II) to implement the web-based survey in SurveyMonkey (SurveyMonkey.com, LLC). Representatives from the national ministry of health or related national organization responsible for vaccine policies were invited to complete the survey. For a full list of VENICE II country gatekeepers, please see http://venice.cineca.org/participating_countries.html. For the non-VENICE II advanced economies, we worked with representatives from the Australia Department of Health and Aging; the Public Health Agency of Canada; The Department of Health Hong Kong; the National Institute of Infectious Disease, Japan; The Korea Centers for Disease Control and Prevention; the University of Otago in New Zealand; and the Taiwan Centers for Disease Control and Prevention. If invited participants did not respond, web searches of publicly available vaccine schedules and policies published by the national ministry of health were performed to fill data gaps.

VENICE II served as the data collectors and managers for the web-based survey, and responded to questions regarding the survey. Countries that completed the web-based survey were asked to validate their data by reviewing their responses and updating them as necessary. Data collection continued through February 2011. For the remaining 11 countries and SARs, we administered the MS Word-based survey via email that contained the same questions as the web-based survey. Data on seasonal influenza, hepatitis B, and HPV vaccines were available from published reports of recent VENICE II surveys in 2008, 2009, and 2010, respectively. (Dorleans et al. 2010; Mereckiene et al. 2008, 2010) For survey respondents that did not provide an estimate for vaccination coverage for hepatitis B, data from published reports were used to supplement the data. (Bader and Egler 2004; Nielsen et al. 2009; Schenkel et al. 2008; Schiller and Euler 2008; van Houdt et al. 2009).

### Analysis

Data from the web- and MS Word-based surveys were aggregated and analyzed using MS Excel 2007. We calculated the total, mean, and median number of vaccines recommended for adults in each advanced economy. For each of the 16 vaccines or components, we examined the groups of adults for which vaccines were recommended, the availability and type of funding, the availability of vaccination coverage estimates, and vaccination coverage for recommended adult vaccines.

We also examined the relationships between adult vaccine policies and characteristics of the advanced economy using bivariate logistic regression analyses. Odds ratios (ORs) were calculated in SAS (SAS Institute Inc. 2008) using the Proc Logistic procedure. The outcomes examined were availability of a comprehensive adult vaccine schedule (yes/no), recommendations for any group of adult to receive vaccination for a particular vaccine or component (yes/no), availability of any funding (public or private) for a recommended adult vaccine or component (yes/no), and availability of a vaccination coverage estimate for a recommended adult vaccine or component (yes/no). Data for the predictor variables were gathered from the World Bank. (The World Bank 2010a, b, c, d) Predictor variables examined were population (as of 2009), GDP per capita (current $US as of 2009), health expenditure per capita (current $US as of 2009), and out-of-pocket health expenditure (% of private expenditure on health as of 2009). For the outcome “Comprehensive Adult Immunization Schedule (yes/no),” one OR was calculated for each of the four continuous predictor variables. For the outcomes “Recommendation (yes/no),” “Any Funding (yes/no),” and “Coverage Estimate Available (yes/no),” meta-analysis was used to produce pooled ORs. First, for each of the four predictor variables, ORs were calculated when data were sufficient for each of 16 vaccines. The separate vaccine ORs were pooled into one OR for each predictor/outcome combination using the R meta-analysis package named metafor. (Viechtbauer 2010).

The coauthors of this study, except for J. Crumly, also published the results of this survey for VENICE II member countries only in a separate paper. (Kanitz et al. 2012) The VENICE II consortium includes European countries not classified as advanced economies that are not included in the present analysis. The VENICE II only analysis also examines the types of vaccine components recommended, funding, and availability of coverage estimates for adult vaccines, but does not analyze the relationships between adult vaccine policies and characteristics of the countries. The current manuscript highlights findings from a group of countries and SARs with comparable economies, whereas the VENICE II only paper reports results for a group of countries sharing a common geographic location.

## Results

Thirty-one of 33 (93.9 %) advanced economies responded to the survey; Israel and Singapore did not provide responses. The data were validated in 15 of 31 (48.4 %) advanced economies (Table 1). The age of adulthood for immunization purposes ranged from 15 (Spain) to 19 (Slovenia) years, with a median of 18 years. Twelve of 31 (38.7 %) advanced economies had a comprehensive adult vaccine schedule which describes the country or SAR’s adult vaccine recommendations in one document. The total number of vaccines or vaccine components recommended for adults ranged from one to 15 with a median of 10. Having a comprehensive adult vaccine schedule was associated with recommending more vaccines for adults [odds ratio (OR) = 1.38, 95 % confidence interval (CI): 1.007–1.891, *p* value 0.0452].

### Descriptives

**Fig. 1:**
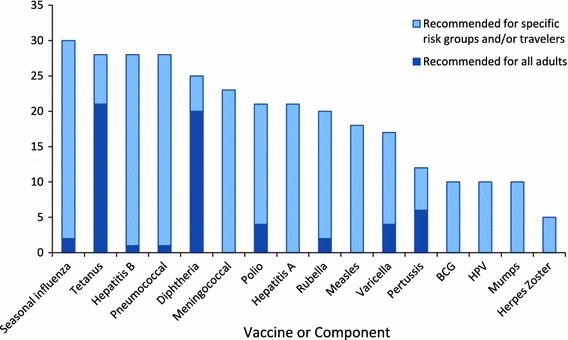
Advanced economies that recommend vaccines for adults, 2010, survey of adult vaccination policies in advanced economy countries (*n* = 30). Specific risk groups = age, health condition, at-risk employee (including health care worker), disabled. Travelers are individuals that travel to disease-endemic areas

Figure 1 shows the number of advanced economies that recommend vaccination for 16 vaccines or components for one or more groups of adults. This figure also indicates whether the recommendations are for all adults or for specific risk groups and travelers to disease-endemic areas. Specific risk groups include recommendations for particular age groups (e.g., older adults), adults with health conditions, at-risk employees including health care workers, and the disabled. For example, 30 (96.8 %) advanced economies recommend adults be vaccinated seasonally against influenza. Of these, two (6.7 %) recommend for all adults and 28 (93.3 %) recommend influenza vaccination for specific groups of adults.

The most frequently recommended vaccines for adults are seasonal influenza (*n* = 30), hepatitis B (*n* = 27), pneumococcal (*n* = 27), tetanus (*n* = 26), and diphtheria (*n* = 26). The least frequently recommended vaccines for adults are Bacille Calmette-Guerin (BCG) (*n* = 10), mumps (*n* = 10), HPV (*n* = 5), and herpes zoster (*n* = 5). Most adult vaccines are recommended for specific risk groups and travelers, with the exceptions of tetanus, diphtheria, and pertussis.

**Fig. 2:**
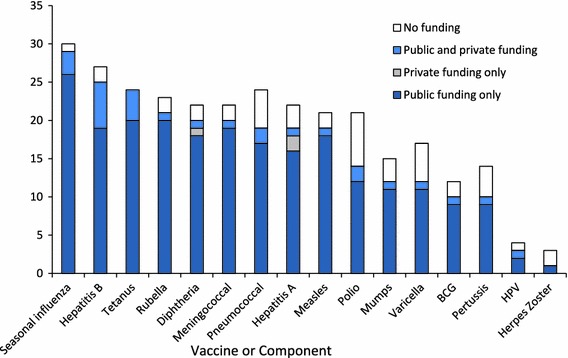
Type of funding mechanisms for recommended adult vaccines, 2010, survey of adult vaccination policies in advanced economy countries (*n* = 30). Private funding = legal requirement for private health insurance to pay for vaccination

Figure 2 shows the type of funding mechanism (public funding only, private funding only, public and private funding, or no funding) for recommended adult vaccines in advanced economies for the same 16 vaccines or components shown in Fig. 1. These funding mechanisms are for one or more groups of adults. Private funding indicates that a legal requirement for private health insurance to pay for vaccination for that particular vaccine or component is in place. As an example, seasonal influenza vaccine is financed through public funds only in 26 of 30 respondents (86.7 %) and through public and private funding in three (10.0 %) advanced economies. Although seasonal influenza vaccine is recommended for adults, no funding is provided for the vaccine in one (3.3 %) advanced economy. Most recommended adult vaccines are financed through public funds only. Pneumococcal (26.3 %), polio (50.0 %), varicella (41.7 %), pertussis (40.0 %), mumps (25.0 %), and HPV (33.3 %) vaccines have no funding mechanism for adults in 25 % or greater of recommending advanced economies.

At least one advanced economy had a coverage estimate available for each of the 16 recommended adult vaccines or components for one or more groups of adults. Seasonal influenza vaccination coverage was the most commonly measured, with 29 (96.7 %) of advanced countries indicating a vaccination coverage estimate for adults is available. After seasonal influenza, the vaccines for which coverage was most commonly measured were hepatitis B [*n* = 11 (42.3 %)] and tetanus [*n* = 8 (30.8 %)]. Six recommending advanced economies each had vaccination coverage estimates for diphtheria (28.6 %) and pneumococcal (35.3 %). Four recommending advanced economies had vaccination coverage estimates for hepatitis A (22.2 %). Three recommending advanced economies had a vaccination coverage estimate for the polio vaccine (15.8 %). One advanced economy had a vaccination coverage estimate available each for varicella (5.9 %), measles (6.3 %), mumps (6.7 %), rubella (6.3 %), meningococcal (6.7 %), pertussis (5.6 %), BCG (7.1 %), HPV (16.7 %), and herpes zoster (25.0 %). (Please note: the Australian Institute of Health and Welfare released the results of the 2009 Adult Vaccination Survey on March 3, 2011. To maintain comparability across the study period March 2010–February 2011, we do not include the results here.)

**Fig. 3:**
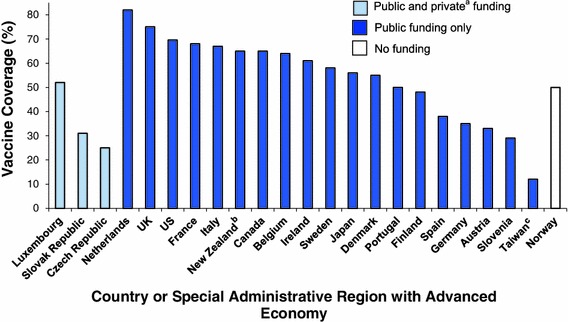
Seasonal influenza vaccination coverage estimates by advanced economy and type of funding for adults ≥65 years, most recent from 2006 to 2010 (*n* = 23) ^a^ Legal requirement for private health insurance to pay for vaccination ^b^ Data for New Zealand from a national serosurvey by the New Zealand Ministry of Health from Oct 2005 to Feb 2007, *n* = 597 ^c^ Data for Taiwan for all adults ≥18 years. Source: Centers for Disease Control and Prevention (2010), Kwong et al. (2007), Mereckiene et al. (2008), Hajime Kamiya (Personal communication)

Figure 3 shows seasonal influenza vaccination coverage for adults ≥65 years for the most recent influenza season for which data are available between 2006 and 2010 for 23 advanced economy countries (the data for Taiwan are for all adults ≥18 years.). The Figure also shows the type of funding mechanism for seasonal influenza vaccine. Vaccination coverage ranged from 82 % in the Netherlands to 25 % in the Czech Republic. Coverage in Taiwan was 12 %. Despite not having any funding for seasonal influenza vaccine for adults, the coverage estimate for adults ≥65 years in Norway (50 %) was higher than in seven advanced economies that do have a funding mechanism for the vaccine. The seasonal influenza vaccination coverage among adults ≥65 years in these seven advanced economies was 31 % in the Slovak Republic, 25 % in the Czech Republic, 48 % in Finland, 38 % in Spain, 35 % in Germany, 33 % in Austria, and 29 % in Slovenia.

Table 2 shows vaccination coverage estimates among adults for tetanus and hepatitis B vaccines for recommending advanced economies where data were available. In six advanced economies, tetanus vaccination coverage ranged from 47 % among all adults (≥18 years) in Canada to 71 % among all adults (≥18 years) in France. The tetanus coverage estimate was 6 % among 16- 24-year-olds in New Zealand and 95 % among 25- 44-year-olds also in New Zealand. However, the estimates in New Zealand were based on a national serosurvey conducted among 597 individuals rather than vaccination coverage measurement. (Weir et al. 2009).

**Table 2:** Tetanus and hepatitis B vaccination coverage estimates for adults in recommending advanced economies, 2010, survey of adult vaccination policies in advanced economy countries

Advanced economy	Type of funding	Vaccination coverage (%)		Years
*Tetanus*				
Canada	Public only	All adults	47	2006
France	Public only	All adults	71	2002
Germany	Public only	All adults	73	2009
Malta	Public only	16 years^a^	55	2009
New Zealand^b^	Public only	16–24 years	6	2007
		25–44 years	95	
		≥45 years	89	
Portugal	Public only	65 years	61	2010
United States	Public only	19–49 years	64	2008
		50–64 years	62	
		≥65 years	52	
*Hepatitis B*, *by age groups*				
Canada	Public only	18–49 years	58	2006
Denmark (Nielsen et al. 2009)	Public only	18–49 years	8	2007
Germany (Bader and Egler 2004)	Public and private^c^	18–49 years	46	2003
		50–59 years	33	
		≥60 years	33	
United States (Schiller and Euler 2008)	Public only	19–49 years, not hr	34	2008
		19–49 years, hr	32	
*Hepatitis B*, *by risk groups*				
Czech Republic	Public and private	HCW	100	2007
Finland	Public only	IDU	45	2004
France	Public only	HCW	87	1999
Germany (Schenkel et al. 2008)	Public only	Chronic condition	70	2004
		HCW	22	
Netherlands (van Houdt et al. 2009)	Public only	MSM, CSW, IDU^d^	62	2002–2007
Slovakia	Public and private	HCW	88	2007
		Hemodialysis patients	96	
United States	Public only	HCW	69	2008

Norway has tetanus vaccination coverage for adults available but did not provide the estimate

*hr* High risk, *HCW* health care workers, *IDU* intravenous drug users, *MSM* men who have sex with men, *CSW* commercial sex workers

^a^Malta recommends a 5th dose of tetanus vaccine at 16 years but the age of adulthood is 18 years

^b^From a national serosurvey by the Ministry of Health from Oct 2005–Feb 2007, *n* = 597

^c^Public and private funding indicates public funding is available and there is a legal requirement for private health insurance to pay for vaccination

^d^From a sample of MSM, CSW, and IDU

*Source* Nielsen et al. (2009); Bader and Egler (2004); Schiller and Euler (2008); Schenkel et al. (2008); van Houdt et al. (2009)

Hepatitis B vaccination coverage is shown by age groups and by risk groups of adults in Table 2. Among younger adults (from the adult age threshold up to 49 years), hepatitis B vaccination coverage ranged from 8 % in Denmark to 58 % in Canada. Among older adults (≥50 years), there is one estimate for Germany at 33 % coverage. Among health care workers, hepatitis B vaccination coverage ranged from 22 % in Germany to 100 % in the Czech Republic. For adults in behavioral risk groups, coverage ranged from 45 % among intravenous drug users (IDU) in Finland to 62 % in a sample of men who have sex with men, commercial sex workers, and IDU in the Netherlands.

### Regression analyses

In Table 3, we show results of bivariate logistic regression analyses for the associations between advanced economy characteristics (population, GDP per capita, health expenditure per capita, and out-of-pocket health expenditure) and adult immunization strategies (adult immunization schedule, recommendation for a particular vaccine or component, any funding for a particular vaccine or component, and coverage estimate available for a particular vaccine or component). We adjusted the scale for population (to population per 5 million), GDP per capita (to GDP per capita per US $5,000), and health expenditure per capita (to health expenditure per capita for US $1,000) so the results would have a practical interpretation. Population per five million was associated with slightly increased odds of having a coverage estimate available. There was a marginally statistically significant reduced odds of having any funding available for the 16 vaccines or components as GDP per capita per US $5,000 increases (pooled OR = 0.94, 95 % CI 0.86–1.03). Both GDP per capita and health expenditure per capita were related to an increased probability of recommendation for vaccines, while increasing out-of-pocket health expenditure was associated with decreased odds of a recommendation for a vaccine and having a coverage estimate available (see Table 3). Recommendation for vaccination was related to having a comprehensive adult immunization schedule (pooled OR = 1.73, CI 1.30–2.29). Please note this finding is not reported in Table 3.

**Table 3:** Results of logistic regression analysis, 2010, survey of adult vaccination policies in advanced economy countries

Predictor^b^	Odds ratio (95 % confidence interval)	Odds ratio (95 % confidence interval) for all 16 vaccines or components^a^		
	Comprehensive adult immunization schedule (yes/no)	Recommendation (yes/no)	Any funding (yes/no)	Coverage estimate available (yes/no)
Per 5 million population	1.08 (0.96–1.22)	1.01 (0.98–1.03)	1.00 (0.95**–**1.06)	**1.05 (1.00–1.09)**
GDP per capita per $US5000	0.92 (0.71–1.18)	**1.10 (1.02–1.19)**	0.94 (0.86–1.03)	0.96 (0.86–1.06)
Health expenditure per capita per $US1000	1.00 (0.64–1.57)	**1.33 (1.16–1.53)**	–	1.06 (0.88–1.27)
Out of pocket health expenditure in percent^c^	0.93 (0.87–1.00)	**0.96 (0.95–0.98)**	1.01 (0.99–1.03)	**0.97 (0.95–0.99)**

^a^Diphtheria, Bacillus Calmette-Guérin (BCG), hepatitis B, hepatitis A, herpes zoster, human papillomavirus (HPV), measles, meningococcal, mumps, pertussis, poliomyelitis (polio), pneumococcal, rubella, seasonal influenza, tetanus, and varicella

^b^Data from the World Bank (see “Methods”)

^c^Percent of total private expenditure on health

^d^Bold: *p* value for test statistic <0.05

## Discussion

This survey is the first to describe the range of adult vaccine strategies, policies, and funding sources in 31 advanced economies. Roughly one-third of the advanced economies have a comprehensive adult immunization schedule that summarizes their recommendations. We found a correlation between having a comprehensive adult immunization schedule and recommending more vaccines for adults. Although the confidence interval was wide, this finding could indicate that the importance of adult vaccination and the perception of the severity of vaccine-preventable diseases may vary from place to place.

We found the most frequently recommended vaccines for adults are seasonal influenza, tetanus, diphtheria, pneumococcal, and hepatitis B. These data are not surprising given that these five vaccines have been widely used for more than 30 years and were more often initially recommended for adults rather than children compared with other vaccines, such as varicella or polio. (Hinman and Orenstein 2007; Michel and Lang 2011; Poland et al. 2010; Roush and Murphy 2007) The least frequently recommended adult vaccines are BCG, HPV, mumps, and herpes zoster. BCG vaccination has varying efficacy against tuberculosis, especially among adults (ACIP 1990), and HPV and herpes zoster are relatively new vaccines. We also found most adult vaccines are recommended for specific groups of adults rather than recommended to all adults, consistent with cost effectiveness and indications for most of these vaccines. The age threshold of adults defined by the surveyed countries ranged from 15 to 19 years, which may affect the comparability of the survey results.

Lowering the financial barriers to receiving vaccines increases vaccine uptake. (The Community Guide 2008) Therefore, an effective funding mechanism for adult vaccines is an important part of a comprehensive immunization strategy. Our results indicate increasing GDP per capita and increasing health expenditure per capita are associated with increased likelihood of a recommendation for a vaccine. We also found the majority of recommended adult vaccines are funded through public funds only. No international system of classifying health systems currently exists, and advanced economies may define public and private funding differently. The type of funding available may also be limited to public only if the advanced economy has a centrally run national health care system, such as the National Health Service in the UK.

Most recommended adult vaccines have a source of funding, whether it is public or private, but it is important to note that pneumococcal, polio, pertussis, varicella, mumps, and HPV vaccines do not have a funding mechanism in more than 25 % of recommending advanced economies. This lack of funding may be related to the length of time the vaccine has been recommended for adults, the epidemiology of disease burden, competing health interests, or the process of making vaccine recommendations in each advanced economy. In the survey, we asked for the year when a vaccine recommendation for adults was first made for each vaccine or component, but the majority of respondents did not provide a response. We did not have enough data to examine the relationship between funding mechanisms and health expenditure per capita. It may be that, as more government funds are spent on health in general, the need to fund adult vaccination is less perceived. However, this finding could be confounded by health care efficiency, as increased spending on health per capita does not necessarily correlate with improved quality of care. (Anderson and Frogner 2008) We did not ask about the processes of making vaccine recommendations in each advanced economy, which could be valuable in understanding the factors influencing adult vaccine policies. These could include deliberations by national or regional vaccine advisory committees, and how often vaccine policies are reviewed and updated. This information could be useful to understanding the decision-making process around vaccines and the relative importance of adult vaccination compared with other health priorities.

Vaccination coverage measurement is vital to evaluating immunization program progress. We found that, for all 16 vaccines or components except for seasonal influenza, the majority of recommending advanced economies do not have a coverage estimate available. The financial and structural resources needed for regular vaccination coverage measurement can be substantial, and adult vaccination may not be a health priority in many of these advanced economies.

We described vaccination coverage estimates for the three vaccines for which we have the most data: seasonal influenza, tetanus, and hepatitis B. Coverage rates vary substantially across similar age or risk groups. For seasonal influenza vaccine among adults 65 years and older, many advanced economies have reached coverage rates above 60 %, with a high of 82 % in the Netherlands. In comparison, a number of advanced economies have coverage below 40 % among the same age group. This may be related to the degree to which regular seasonal influenza vaccination is a long-standing part of preventive care.

We received responses from over 90 % of the invited respondents, and while the data are based on self-report, these were validated in roughly half of the respondents. Some respondents expressed confusion about the meaning of a vaccine “recommendation,” and we received some comments that a vaccine recommendation could be made by a range of groups, including the government or Ministry of Health and also by health care provider associations and vaccine manufacturers. Because this was a survey of adult vaccine policies, we asked about vaccines recommended by the government, Ministry of Health, or another official policy-making body in the advanced economy. We did not ask about the vaccine safety systems in these advanced economies, which is another important component of a vaccination program.

While ongoing vaccine uptake measurement can be resource intensive, guidelines to establishing such systems may be helpful for these advanced economies. A consensus statement issued by the European Union Geriatric Medicine Society and International Association of Gerontology and Geriatrics-European Union in 2009 advocated for strengthening and harmonizing vaccine strategies for adults 60 years and older at the European level. (Michel et al. 2009) Policies and programs are important not only for older adults, but for all adults to protect the well-being and health of the entire population. Advanced economies are experiencing aging of their populations, and with continuing improvements in medicine and the quality of life, public health will need to consider a shift in funding from childhood-based vaccination programs to “lifespan” programs. (Michel and Lang 2011; Poland et al. 2010).

The reduced effectiveness of some adult vaccines when compared with childhood vaccines may be a barrier to increased uptake. (Osterholm et al. 2012) As the number of available vaccines continues to increase, standards for vaccination strategies will ease the introduction of new vaccines into existing vaccination practices. An increasing role exists for non-traditional/non-medical home immunization providers, such as pharmacists and community vaccinators, and standards should include considerations for partnering with these health providers. (Postema and Breiman 2000).

### Conclusions

We have demonstrated policies recommending vaccination for adults, funding mechanisms for adult vaccine administration, and routine adult vaccination coverage assessment vary and are often lacking in the countries with advanced economies surveyed. Newer vaccines are less likely to be recommended for adults than older ones. Funding of adult vaccination is associated with recommendations for adult vaccine use. Most recommended adult vaccines are funded with public funds alone. These elements of stable funding, standard recommendations, and routine vaccine coverage assessment are essential components of successful childhood immunization programs. For adult vaccination to be as effective as childhood programs, countries should strive to include them for their adult populations.

## Electronic supplementary material

Below is the link to the electronic supplementary material.

Supplementary material 1 (DOCX 18 kb)
