# Supplementary material for: Adult immunization policies in advanced economies: vaccination recommendations, financing, and vaccination coverage
Source: Int J Public Health. 2013 Jan 25;58(6):865–74. doi: 10.1007/s00038-012-0438-x (PMC3840285; doi:10.1007/s00038-012-0438-x)
Supplement: Supplementary file 1 — Supplementary material 1 (DOCX 18 kb) [file 38_2012_438_MOESM1_ESM.docx]

**Supplemental Table 1. Advanced economies by adult vaccine recommendations, funding type, and availability of coverage estimates, 2010, survey of adult vaccination policies in advanced economy countries**

| **Vaccine or Component** | **Recommendation** | | | **Required Funding for Recommended Adult Vaccines for One or More Groups of Adults** | | | | **Adult Coverage Estimate Available** |
| --- | --- | --- | --- | --- | --- | --- | --- | --- |
|  | **For All Adults** | **For Specific Risk Groups**  **(e.g., age, disabled, occupational risk)** | **For Travelers to Disease-endemic Countries** | **Public Only** | **Private Only** | **Public and Private** | **None** |  |
| **BCG** |  | AU, FR, IR, IT, MT, NO, SK, UK | DK, NL | CY, FR, IR, IT, LU, MT, NO, SK, SD, UK |  | SK | IC, NL | MT |
| **Diphtheria** | AT, BE, CA, CY, DK, FI, FR, GE, GR, IT, LU, NZ, NO, PT, SK, SL, SP, SW, TW,US | AU, MT, UK | BE, FR, IC, IR MT, NL, SP, UK | AU, BE, CA, CY, DK, FI, FR, GE, GR, IR^b^, IT, LU, MT, NZ, PT, SL, SD, US | NL | SK, SP | IC, NO | BE, FR, MT, NZ, PT US |
| **Hepatitis A** |  | AT, CA, CY, CZ, DK, FR, GE, GR, IR, LU, NL, NZ, NO, SK, SL, SW, TW, US | AT, BE, CY, DK, GE, GR, IC, IR, JP, NL, NZ, SL | BE, CY, CZ, DK, FI, GE, GR, IR, IT, MT, NO, SL, SP, SD, UK, US | LU, NL | SK | FR, IC, NZ | CA, HK, NZ, US |
| **Hepatitis B** | AT | AT, BE, CA, CY, CZ, DK, FI, FR, GE, GR, HK, IC, IR, IT, LU, MT, NL, NZ, NO, PT, SK, SL, SP, SD, SW, TW, UK, US | BE, CY, DK, FI, FR, GE, IR, IT, LU, NL, NO, PT, SL, SP,UK | CA, CY, DK, FI, FR, HK, IC, IR, IT, MT, NL, NO, SL, SP, SD, TW, UK, US |  | BE, CZ, GE, PT, SK | AU, GR | AU, CA, CZ, DK, FI, FR, GE, NL, NZ, SK, US |
| **Herpes zoster** |  | AT, BE, LU, TW,US |  | US |  |  | FI, SD | US |
| ***Haemophilus influenzae* Type b (Hib)** |  | GE, IR, PT, SP, |  |  |  |  |  |  |
| **HPV** |  | AT, BE, FR, GR, IT, JP, NL, SW, TW,US |  | FR, UK |  | GE | US | US |
| **Japanese Encephalitis** |  |  | NL |  |  |  |  |  |
| **Measles** |  | BE^a^, CA, CY^a^, DK, FI, FR^a^, GE^a^, GR, IR^a^, IT, NZ, PT, SL, SP, TW^a^, UK, US | AT, CA, DK, IR, PT, SP | BE, CA, CY, DK, FI, FR, GE, GR, IR^b^, IT, LU, MT, NZ, PT, SD, SL, UK, US |  | SP | IC, NO | NZ |
| **Meningococcal** |  | CA, CY, CZ, FI, FR^a^, GE, GR, IR, LU^a^, NZ, NO, PT, SK, SL, SP, SW, UK, US | AT, BE, CY, DK, FR, GE, GR, IR, NL, NZ, SL, SP, TW | CY, CZ, DE, FI, FR, GE, GR, IC, IR, IT LU, MT, NO, PT, SL, SP, SD, UK, US |  | SK | BE, CA | IR |
| **Mumps** |  | CA, CY, FI, GE, IR^a^, NZ, SL, TW, UK^a^, US | CA | CA, CY, FI, GE, IR, IT, LU, MT, NZ, SD, US |  | SP | IC, NO, SL | NZ |
| **Pertussis** | AT, CA, GE, LU, NO, TW | AU, BE, FR, SL, SP, US |  | AU, BE, CA, FI, FR, GE, LU, SD, US |  | SP | IC, MT, NO, SL | US |
| **Pneumococcal** | TW | AU, AT, BE, CA, CY, CZ, DK, FI, FR, GE, GR, HK, IC, IR, LU, NL, NZ, NO, PT, SK, SL, SP, SD, SW, UK, US |  | AU, CA, CY, FR, GE, GR, HK, IR, LU, MT, NZ, NO, SP, SD, TW, UK,, US |  | CZ, SK | BE, DK, FI, IC, PT, SL | CA, IR, TW, US |
| **Polio** | AT, FR, LU, NZ | AT, CA, CY, DE, GE, GR, IC, IR, MT, NZ, PT, UK | BE, CA, FI, FR, GE, GR, IC, IR, MT, NL, NO, NZ, PT, SL, SP, TW, UK | CY, DK, FI, FR, GE, GR, IR, IT, LU, PT, SL, SD |  | MT, SP | BE, CA, IC, NL, NZ, NO, US | FR, MT, NZ |
| **Rabies** |  | AT, CY, FI, FR, GE, IR, IT, LU, NL, NO, PT, SK, SL, SP, UK | AT, BE, CY, DK, FI, FR, GE, IC, IR, NL, SP, UK |  |  |  |  |  |
| **Rubella** | CA, TW | AT, BE, DK, FI, FR, GE, GR, HK, IC, IR, IT, NZ, NO, PT, SL, SP, SD, US |  | BE, CA, CY, DK, FI, FR, GE, GR, HK, IC, IR, IT, LU, MT, NZ, NO, PT, SD, TW, US |  | SP | NL, SL | NZ |
| **Seasonal influenza** | AU, SK,TW, US | AU, AT, BE, CA, CY, CZ, DK, FI, FR, GE, GR, HK, IC, IR, IT, JP, KO, MT, NL, NZ, NO, PT, SK, SL, SP, SD, SW, UK |  | AU, AT, BE, CA, CY, DK, FI, FR, GE, GR, HK, IC, IR, IT, JP, KO, MT, NL, NZ, PT, SL, SP, SD, TW, UK, US |  | CZ, LU, SK | NO | AT, BE, CA, CZ, DK, FI, FR, GE, IR, IT, JP, LU, NL, NZ, NO, PT, SK, SL, SP, SD, TW, UK, US |
| **Tick-borne Encephalitis (TBE)** |  | GE, SL | NL |  |  |  |  |  |
| **Tetanus** | AT, BE, CA, CY, CZ, DK, FI, FR, GE, GR, IT, LU, NZ, NO, PT, SK, SL, SP, SW, TW, US | AU, CY, FI, MT, NL, SP, TW, UK | AU, BE, FR, IR, MT, NL, SP, UK | AU, BE, CA, CY, DK, FI, FR, GE, GR, IC, IR^b^, IT, LU, MT, NZ, NO, PT, SL, SD, US |  | CZ, NL, SK, SP |  | BE, CA, FR, GE, MT, NO, NZ, PT, US |
| **Varicella** | BE, CA, NZ, US | AT, FI, FR, GE, IR, IT, LU, NL, SL, SP, SW, TW, UK | SW | CY, FI, FR, GE, IR^b^, IT, LU, MT, SL, SD, US |  | SP | CA, IC, NL, NO, NZ | NZ |

^a^ Catch-up recommendation if did not complete childhood series

^b^ In some regions of the advanced economy country
